# Triglyceride Glucose Index and Stress Hyperglycemia are Important Factors for Predicting Early Neurological Deterioration in Isolated Acute Pontine Infarction

**DOI:** 10.1002/brb3.70746

**Published:** 2025-08-04

**Authors:** Jian Ge, Chunjie Song, Yuanyuan Han

**Affiliations:** ^1^ Department of Neurology Jiangsu Province (Suqian) Hospital Suqian Jiangsu China

**Keywords:** early neurological deterioration, pontine infarction, stress hyperglycemia, triglyceride‐glucose

## Abstract

**Objective:**

The triglyceride‐glucose (TyG) index and stress hyperglycemia (quantified as fasting plasma glucose/glycosylated hemoglobin ratio, GAR) are strongly associated with acute ischemic stroke (AIS) progression and adverse clinical outcomes. However, the relationship between the TyG index, GAR, and early neurological deterioration (END) in isolated acute pontine infarction (IAPI) patients remained undefined. The aim of this study was to investigate the diagnostic value of the TyG index and GAR for END in patients with IAPI.

**Methods:**

This study enrolled 192 patients diagnosed with IAPI at Jiangsu Province (Suqian) Hospital between March 2022 and June 2024. Patients were stratified into END and non‐END groups based on National Institutes of Health Stroke Scale (NIHSS) criteria. Clinical data and hematological indicators of the two groups were collected, and the TyG index and GAR were calculated. Risk factors for END were analyzed by multifactorial logistic regression. The diagnostic value of the risk factors was assessed by receiver operating characteristic (ROC) curves.

**Results:**

Among 192 participants, 53 (27.6%) developed END. Significant intergroup differences were observed in baseline NIHSS scores, IAPI etiological subtypes, TG, LDL‐C, FPG, TyG index, and GAR (all *p* < 0.05). The TyG index and GAR were risk factors for END by multivariate logistic analysis (all *p* < 0.05). ROC analysis revealed AUC values of 0.769 (95% CI: 0.657–0.841, *p* < 0.001) for the TyG index and 0.752 (95% CI: 0.632–0.826, *p* < 0.001) for GAR in predicting END. The optimal cut‐off values were 8.52 and 18.72. The area under the curve of the combined prediction END of the TyG index and GAR was 0.839 (95% CI: 0.728‐0.882, *p* < 0.001).

**Conclusion:**

Elevated TyG index and GAR serve as independent risk factors for END in IAPI patients, demonstrating significant diagnostic potential as combined biomarkers.

## Graphical abstract

Branch atheromatous disease (BAD) is the primary etiology of isolated acute pontine infarction (IAPI) and is associated with the highest risk of early neurological deterioration (END). Elevated TyG index and stress hyperglycemia (quantified as fasting plasma glucose/glycosylated hemoglobin ratio, GAR) act as independent risk factors for END in IAPI patients, demonstrating significant diagnostic potential as combined biomarkers.

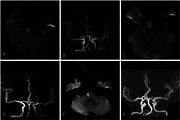

## 1 Introduction

Isolated acute pontine infarction (IAPI) represents one of the most prevalent subtypes of posterior circulation acute ischemic stroke (AIS), constituting approximately 15% of posterior circulation AIS cases (Bi et al. 2021). The primary pathological mechanisms involve parent artery atherosclerosis and perforating artery disease (Huang et al. 2016). During the acute phase, 14–35% of IAPI patients experience early neurological deterioration (END), predominantly manifesting as motor deficits (Oh et al. 2012). END exacerbates physical disability and is strongly correlated with adverse clinical outcomes. While the precise pathophysiology of END remains incompletely understood, proposed mechanisms encompass local thrombus propagation, vasogenic edema, blood‐brain barrier breakdown, excitotoxicity, and neuroinflammation (Liu et al. 2022).

Diabetes mellitus and insulin resistance (IR), hallmarks of metabolic syndrome, constitute predominant risk factors for cerebrovascular pathologies (Qu et al. 2024). Chronic hyperglycemia drives progressive vascular complications through sustained endothelial dysfunction. The triglyceride‐glucose (TyG) index, derived from fasting triglyceride and glucose levels, serves as a validated surrogate marker of IR and predicts unfavorable outcomes in AIS patients (Liu et al. 2024; Ma et al. 2022).

Stress‐induced hyperglycemia manifests in both diabetic and nondiabetic populations during acute physiological stress. It is associated with increased END and infarct volume in AIS (Capes et al. 2001). Conventional metrics such as random or fasting plasma glucose fail to contextualize glycemic status relative to baseline control. Glycated hemoglobin can reflect the average blood glucose level over the past 2–3 months. The fasting plasma glucose‐to‐glycated hemoglobin ratio (GAR) demonstrates superior predictive utility for acute disease progression compared to absolute hyperglycemic thresholds (Roberts et al. 2015). Nevertheless, the prognostic significance of the TyG index and GAR for END in IAPI remains underexplored. This study aims to elucidate the independent associations between elevated TyG index, GAR, and END development in IAPI cohorts.

## 2 Methods

### 2.1 Study Population

This observational study consecutively enrolled patients diagnosed with IAPI at Jiangsu Province (Suqian) Hospital between March 2022 and June 2024. Written informed consent was obtained from all participants or legally authorized representatives prior to data collection. Inclusion criteria: (1) MRI‐confirmed IAPI with symptom‐to‐admission interval ≤72 h; (2) Age ≥ 18 years old; (3) Comprehensive cerebrovascular evaluation via MR angiography or CT angiography; Exclusion criteria: (1) Concomitant anterior circulation infarction; (2) Cardiogenic stroke with evidence of embolic origin; (3) History of severe stroke (Modified Rankin Scale [mRS] score ≥ 2); (4) Patients with thrombolysis and /or bridging thrombolysis; (5) Active malignancies, hematologic disorders, or severe organ dysfunction (cardiac, pulmonary, hepatic, or renal); (6) Acute/chronic infections (e.g., respiratory tract, urinary tract, or gastrointestinal) within 14 days preceding enrollment.

### 2.2 Data Collection

Baseline parameters collected at admission included: demographic characteristics and medical history; blood pressure measurements (systolic/diastolic). Stroke severity assessments: National Institutes of Health Stroke Scale (NIHSS) and mRS; Fasting (8–10 h) venous blood samples analyzed for: glycated hemoglobin (HbA1c) and fasting plasma glucose (FPG); Lipid profile: triglycerides (TG), total cholesterol (TC), high‐density lipoprotein (HDL) and low‐density lipoprotein (LDL); Stress hyperglycemia was quantified using the GAR, calculated as GAR = FPG (mg/dL) / HbA1c (%). The TyG index was derived using “ln [fasting blood glucose level (mg/dL) × triglyceride level (mg/dL)] /2.”

### 2.3 Image Evaluation

**FIGURE 1:**
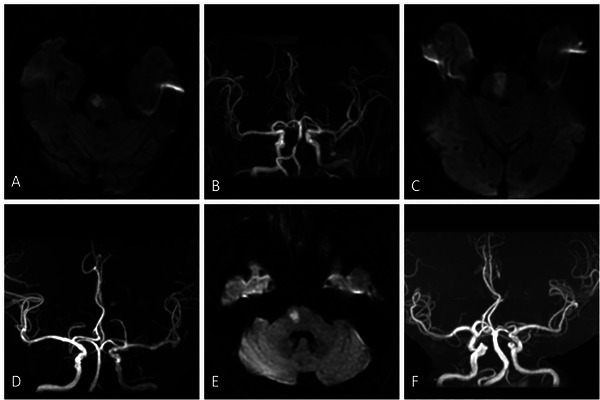
Etiological subtypes of IAPI. A‐B: Large‐artery atherosclerosis: A: Right pontine median infarction; B: MRA showed severe basilar artery stenosis (≥ 50%); C‐D: Branch atheromatous disease: C: Acute infarction of the right pontine reaches the ventral cerebral surface of the pontine, located on one side without crossing the midline; D: MRA showed no significant stenosis of the vertebrobasilar artery (< 50%); E‐F: Small vessel occlusion: E: Right pontine infarction; the infarction lesion does not reach the ventral brain surface of the pontine; F: MRA showed no significant stenosis of the vertebrobasilar artery (< 50%).

All patients received standardized neuroimaging (MRI or CT angiography) within 48 h post‐admission. Etiologic classification followed established neurovascular criteria. Patients with IAPI were classified into three etiologic subtypes (Enriquez‐Marulanda et al. 2016; Kim and Do 2023): (1) Large artery atherosclerosis (LAA): Basilar artery stenosis ≥ 50%; (2) Branch atheromatous disease (BAD): Pontine infarct extending to ventral surface, absence of significant vertebrobasilar stenosis (< 50%), and exclusion of alternative etiologies; (3) Small vessel disease (SVD): Non‐ventral surface pontine infarct, vertebrobasilar stenosis <50%, and without identifiable embolic source. (Figure 1)

### 2.4 Neurological Evaluation and END Classification

Stroke severity was longitudinally evaluated using the NIHSS by two board‐certified vascular neurologists. Discrepancies in NIHSS scoring were resolved through consensus review by a senior neurologist. Patients were categorized into END and non‐END groups according to whether the NIHSS score increased by ≥ 2 points or ≥ 1 point in motor function score within 7 days of onset (Enriquez‐Marulanda et al. 2016).

### 2.5 Statistical Analysis

Statistical analyses were performed using SPSS Statistics 27.0 (IBM Corp., Armonk, NY). Normally distributed continuous variables are presented as mean ± standard deviation (SD) and analyzed with Student's *t*‐test. Non‐normally distributed variables are expressed as median (interquartile range, IQR) and compared using Mann–Whitney *U* tests. Categorical variables are reported as frequencies (%) and analyzed with χ^2^ tests. Multivariable logistic regression analysis identified END risk factors, with results expressed as adjusted odds ratios (OR) and 95% confidence intervals (CI). Fasting plasma glucose (FPG) and triglycerides (TG) were excluded from the regression model to mitigate multicollinearity. Diagnostic performance was evaluated through receiver operating characteristic (ROC) curve analysis, with area under the curve (AUC) quantifying discriminative capacity. The Youden index (J) was determined using J = Sensitivity + Specificity ‐ 1. Statistical significance was defined as two‐tailed *p* < 0.05.

### 2.6 Ethics Approval

The study was conducted in accordance with the Declaration of Helsinki and approved by the Jiangsu Province (Suqian) Hospital Ethical Review Committee (NO.2024‐SL‐0206). All data were fully anonymized before statistical analysis.

## 3 Results

### 3.1 Baseline Characteristics

Among 192 enrolled patients, 53 (27.6%) experienced END. Significant intergroup differences were observed in baseline NIHSS scores, etiological subtypes, triglycerides (TG), low‐density lipoprotein cholesterol (LDL‐C), fasting blood glucose (FBG), TyG index, and glucose‐to‐HbA1c ratio (GAR) (*p* < 0.05). No significant differences were detected in other baseline parameters, including demographic characteristics and comorbidities. (Table 1).

**TABLE 1:** Comparison of baseline data between two groups of patients.

Variable	Non‐END (*n* = 139)	END (*n* = 53)	*p*‐value
Age (years)	64 (56,75)	66 (58,77)	0.472
Male (*n*, %)	86 (61.9)	34 (66.0)	0.770
BMI (kg/m^2^)	23.82 (21.96, 25.37)	25.45 (22.54, 27.88)	0.068
Hypertension (*n*, %)	92 (66.2)	37 (69.8)	0.633
Diabetes (*n*, %)	51 (36.7)	27 (50.9)	0.072
Coronary disease (*n*, %)	34 (24.5)	15 (28.3)	0.585
Smoking (*n*, %)	54 (38.8)	24 (45.3)	0.417
Take hypoglycemic medications (*n*, %)	40 (28.8)	13 (24.5)	0.556
Take lipid‐lowering medications (*n*, %)	22 (15.8)	6 (11.3)	0.429
Etiological classification (*n*, %)			< 0.001
SVD (*n*, %)	52 (37.4)	5 (9.4)	
LAA (*n*, %)	39 (28.1)	20 (37.7)	
BAD (*n*, %)	48 (34.5)	28 (52.8)	
Blood pressure at admission			
SBP, mmHg	156.8 ± 14.9	159.4 ± 14. 1	0.626
DBP, mmHg	88.9 ± 11.5	90.4 ± 10.6	0.583
NIHSS score	3 (2,4)	5 (3,5)	0.008
Laboratory test			
TC (mmol/L)	4.72 (3.82, 5.12)	4.82 (3.91, 5.27)	0.239
TG (mmol/L)	1.43 ± 0.57	1.64 ± 0.65	0.034
LDL‐C (mmol/L)	2.67 ± 0.78	3.06 ± 0.84	0.003
HDL‐C (mmol/L)	1.17 (0.98, 1.32)	1.21 (1.04, 1.36)	0.261
FPG(mg/dL)	6.87 ± 2.22	7.35 ± 2.86	0.017
HbA1c (%)	6.47 ± 1.52	6.58 ± 1.92	0.367
TyG index	8.66 ± 0.65	9.15 ± 0.87	<0.001
GAR	16.38 ± 2.87	21.42 ± 2.58	<0.001

**Abbreviations**: BMI, body mass index; DBP, diastolic blood pressure; FPG, fasting plasma glucose; HbA1c, Glycosylated Hemoglobin, Type A1C; HDL, high‐densitylipoprotein; LDL, low‐densitylipoprotein; NIHSS, National Institutes of Health Stroke Scale; SBP, systolic blood pressure; TC, total cholesterol; TG, triglycerides.

### 3.2 Identification of Independent Risk Factors for END via Multivariable Logistic Regression

Variables demonstrating significant associations with END in univariate analyses (*p* < 0.05) were entered into the multivariable logistic regression model. Diabetes status was forced into the final model regardless of statistical significance. Following adjustment for potential confounders (including age and gender), LDL‐C, GAR, TyG index, LAA, and BAD retained statistical significance as independent predictors of END. (Table 2)

**TABLE 2:** Results of multivariate Logistic regression analysis of END.

	*β*	SE	OR	95% CI	*p* value
NIHSS score	0.452	0.169	1.062	0.884‐1.325	0.278
Diabetes	0.587	0.248	1.526	0.913‐1.875	0.342
LDL‐C	0.227	0.193	2.168	1.583‐4.391	0.024
TyG index	1.313	0.622	3.396	1.654‐11.184	< 0.001
GAR	1.196	0.541	2.214	1.487‐8.168	< 0.001
Etiological classification					
LAA	1.037	0.462	1.533	1.021‐3.796	0.011
BAD	1.291	0.572	3.364	1.933‐8.674	< 0.001

### 3.3 Diagnostic Performance of TyG Index and GAR for END

**FIGURE 2:**
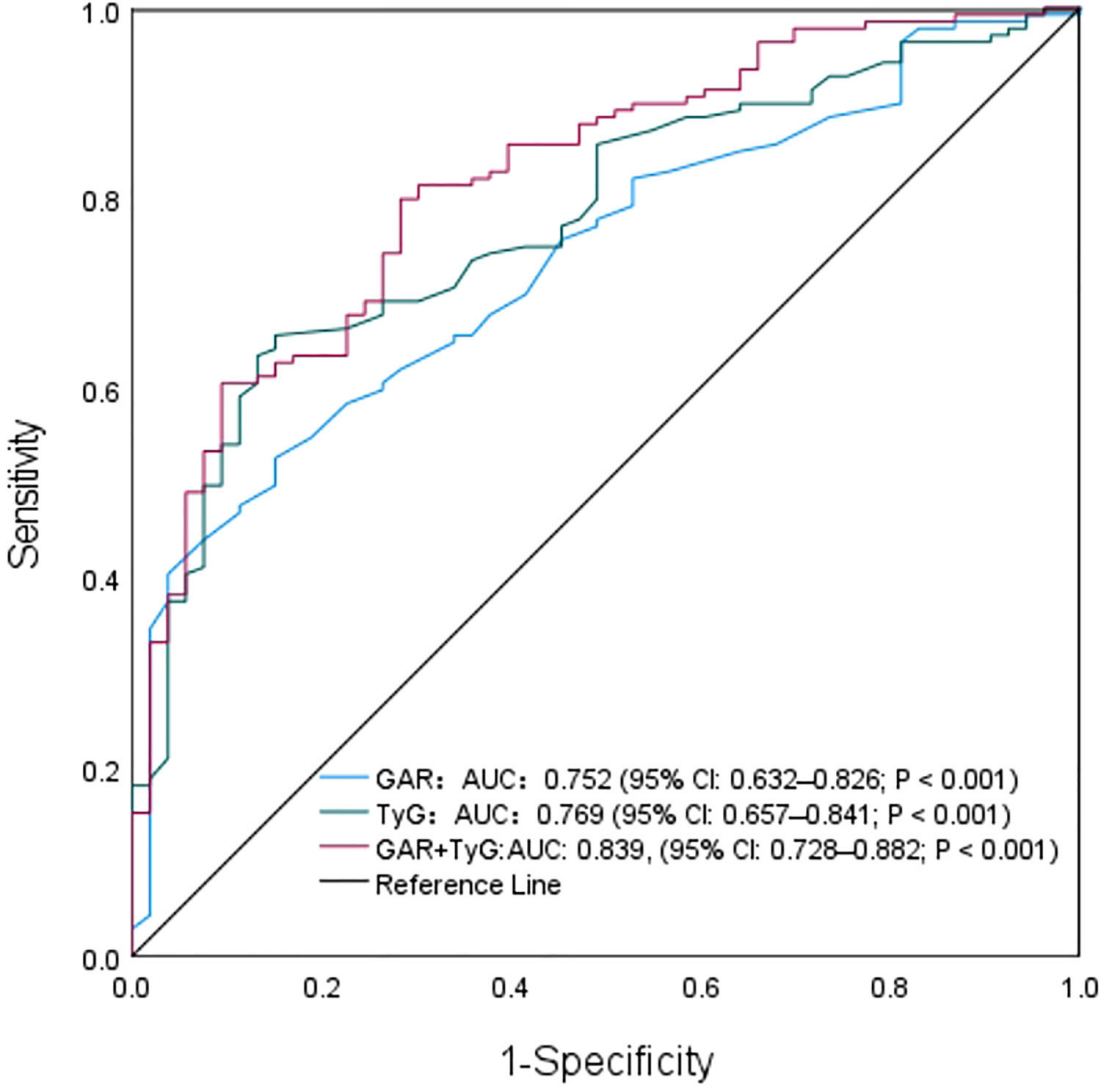
ROC curves for the TyG index and GAR on the diagnostic performance of END.

ROC analysis demonstrated that the TyG index achieved an area under the curve (AUC) of 0.769 (95% CI: 0.657–0.841; *p* < 0.001) for predicting END. The optimal TyG index cutoff value was 8.52, yielding 74.2% sensitivity and 80.3% specificity. GAR showed comparable diagnostic capacity with an AUC of 0.752 (95% CI: 0.632–0.826; *p* < 0.001). At the optimal GAR threshold of 18.72, sensitivity and specificity reached 80.8% and 78.6%, respectively. The combined model integrating the TyG index and GAR demonstrated superior discriminative power (AUC = 0.839, 95% CI: 0.728–0.882; *p* < 0.001). (Figure 2)

Pairwise comparison using DeLong's test revealed significant differences in diagnostic performance between the biomarkers. Specifically, the combined model outperformed both individual markers (both *p* < 0.05), whereas no statistically significant difference was observed between the TyG index and GAR alone (*p* = 0.382). (Table 3)

**TABLE 3:** DeLong's test was used to compare the AUC of TyG, GAR, and TyG + GAR.

	Z	*p* value	95% *CI*
TyG + GAR vs. TyG	2.154	0.013	0.025 ‐ 0.142
TyG + GAR vs. GAR	2.802	0.005	0.043 ‐ 0.232
GAR vs. TyG	0.785	0.382	−0.052 ‐ 0.136

## 4 Discussion

Prognostic stratification in ischemic stroke remains complex due to the heterogeneity in clinical presentations at onset and the distinct pathophysiological features of anterior versus posterior circulation strokes. Similar to basal ganglia infarctions, motor hemiparesis represents the predominant clinical feature in pontine infarction (Maeshima et al. 2012). High‐density white matter tracts in the pons region can easily cause rapid deterioration of the patient's nervous system, often developing into severe disability and adverse clinical outcomes. In our cohort of 192 IAPI cases, BAD constituted the predominant etiology (39.6%), exceeding the prevalence of both LAA (30.7%) and SVD (29.7%). This etiological distribution pattern aligns with prior reports emphasizing BAD as the principal mechanism in pontine infarction (Yamamoto et al. 2010; Gökçal et al. 2017). BAD was first proposed by Caplan in 1989 and described as a parapontine arterial blood supply area with infarct lesions that reach the ventral surface of the pontine, and without evidence of large artery stenosis (≥ 50%) or cardiogenic embolism (Enriquez‐Marulanda et al. 2016; Caplan 1989). According to whether the NIHSS score increased by ≥ 2 points or the motor function score increased by ≥ 1 point within 7 days of onset, our patients were divided into END and non‐END. END incidence reached 27.6% (53/192), with BAD patients demonstrating the highest progression rate (36.8%, 28/76), followed by LAA (33.9%, 20/59), both significantly exceeding SVD cases.

Emerging evidence indicates a higher prevalence of diabetes mellitus in posterior circulation stroke cohorts compared to anterior circulation counterparts, potentially attributed to heightened metabolic vulnerability of the vertebrobasilar system to hyperglycemia (Çoban et al. 2015; Palacio et al. 2014). Diabetic vasculopathy preferentially promotes plaque calcification and destabilization in the vertebrobasilar system. Histopathological analyses further reveal that these plaques exhibit a significantly higher rupture risk than carotid plaques (Takahashi et al. 2017). This pathophysiological interplay warrants enhanced monitoring for posterior circulation events in diabetic patients, especially those presenting with pontine infarction. Notably, the pontine infarction cohort demonstrated a higher prevalence of diabetes compared to non‐pontine infarction patients (42.7% vs. 31.4%; *P* < 0.05) (Zhu et al. 2022). Our study demonstrated comparable findings, with 40.6% (78/192) of IAPI patients exhibiting comorbid diabetes mellitus. Therefore, control of risk factors has a significant positive effect on stroke prevention. Despite advancements in cerebrovascular risk factor identification and evidence‐based interventions, measures have reduced the incidence of stroke, but significant challenges persist. Current models predict 3.4 million incident strokes annually among adults ≥18 years by 2030, representing a 20.5% increase from the 2012 baseline (Virani et al. 2020). These projections underscore the critical need for novel biomarker discovery and implementation of precision prevention strategies targeting modifiable vascular risk factors.

Dysregulated glucose metabolism constitutes a key driver of atherogenesis through multiple pathological pathways. Hyperglycemia induces endothelial dysfunction via multiple mechanisms: (1) impaired nitric oxide (NO) bioavailability, (2) enhanced thromboxane A2 (TXA2)‐mediated vasoconstriction, (3) oxidative stress driven by excessive reactive oxygen species (ROS) generation, and (4) release of proinflammatory cytokines such as IL‐6 and TNF‐α. Collectively, these mechanisms disrupt vascular homeostasis and accelerate atherosclerotic plaque formation (Yao et al. 2023; Tremblay et al. 2014; Lemkes et al. 2010). While hyperglycemia directly promotes atherogenesis, IR, the hallmark metabolic defect in diabetes, independently drives vascular inflammation and plaque progression through adipokine dysregulation and ectopic lipid deposition (Scott et al. 2024). IR accounts for 58–72% of excess cardiovascular risk in diabetic populations, as demonstrated by Mendelian randomization studies (Kosmas et al. 2023). IR is pathophysiologically defined as impaired insulin‐mediated glucose disposal in skeletal muscle, adipose tissue, and liver. The 2021 AHA/ASA guidelines highlight that 30% of AIS patients exhibit pre‐diabetes, while 50% of non‐diabetic AIS cases demonstrate IR (Kleindorfer et al. 2021). IR is not only present in diabetic patients but also very common in non‐diabetic patients with AIS (Mi et al. 2020). Even after the stress state has disappeared, this abnormality of glucose characteristics still exists and may exacerbate nerve damage, leading to a poor prognosis. In addition, IR itself is also an important risk factor for stroke, which can increase the risk of stroke recurrence (Zhou et al. 2023). Therefore, these remind us that timely detection of IR can provide additional avenues for early prevention and reduce the occurrence of stroke and adverse events after stroke.

The TyG index, a validated surrogate marker of IR, has been consistently associated with increased mortality risk and adverse clinical outcomes across multiple cohorts (Ma et al. 2022; Zhou et al. 2020; Lee et al. 2021). Previous studies have demonstrated a dose‐dependent relationship between TyG index elevation and acute ischemic stroke (AIS); furthermore, an elevated TyG index has been identified as an independent risk factor for AIS (Feng et al. 2022). In our cohort of 156 IAPI cases, TyG index levels were markedly elevated in patients developing END compared to non‐END counterparts. Multivariable logistic regression confirmed the TyG index as an independent predictor of END, demonstrating a significant dose‐response relationship.

Stress hyperglycemia, defined as transient plasma glucose elevation irrespective of diabetes status, independently predicts in‐hospital mortality and unfavorable functional outcomes in AIS patients (Pan et al. 2017). In alteplase‐treated AIS patients, stress hyperglycemia exerts differential prognostic effects: non‐diabetics with hyperglycemia demonstrate higher disability risk compared to normoglycemic counterparts, exceeding risks observed in diabetic populations (Merlino et al. 2022). Studies on acute coronary syndromes have demonstrated that hyperglycemic patients face nearly double the relative risk of hospitalization and short‐term mortality compared to normoglycemic patients (Capes et al. 2000). Furthermore, a meta‐analysis confirms that stress hyperglycemia independently predicts mortality in AIS (Snarska et al. 2017). The GAR index is based on the glycated hemoglobin of fasting plasma glucose and background blood glucose, which can reflect the stress glucose increase in different background states. Previous studies have shown that GAR is associated with poor prognosis and an increased 1‐year risk of all‐cause mortality in patients with AIS receiving intravenous thrombolysis or endovascular therapy (Merlino et al. 2024; Chen et al. 2019). These findings position stress hyperglycemia as both a marker of acute physiological stress and a mediator of chronic vascular complications through persistent endothelial dysfunction and inflammasome activation (Merlino et al. 2023). Our study also suggested that GAR is a risk factor for END in patients with IAPI.

Potential mechanisms of chronic hyperglycemia leading to END include: (1) Chronic hyperglycemia activates the hypothalamic‐pituitary‐adrenal axis, elevating circulating catecholamines (adrenaline/noradrenaline), glucocorticoids (cortisol), and proinflammatory cytokines (IL‐6, TNF‐α). These hormones can stimulate hepatic gluconeogenesis through upregulation and inhibit peripheral glucose uptake. Paradoxically, cytokine‐mediated overexpression of GLUT1 in brain endothelial cells exacerbates brain glucose influx, leading to lactate accumulation and ischemic penumbra expansion (Dungan et al. 2009; Wang et al. 2022). (2) Oxidative stress‐mediated blood‐brain barrier disruption: reactive oxygen species are produced through oxidase activation; it degrades tight junction protein (ZO‐1), induces matrix metalloproteinase‐9 (MMP‐9) secretion, and increases vascular permeability. This cascade eventually leads to vasogenic edema and hemorrhagic transformation risk (Yuan et al. 2021). (3) Fibrinolytic system dysfunction occurs as hyperglycemia upregulates PAI‐1 expression, thereby diminishing rt‐PA thrombolytic efficacy, prolonging clot dissolution, and promoting secondary thrombus formation (Wang et al. 2023). (4) Endothelial dysfunction: it can lead to elevated von Willebrand factor, increased platelet glycoprotein expression, and enhanced platelet endothelial adhesion. This prothrombotic environment significantly increases the risk of microvascular occlusion (Batiha et al. 2022). (5) Hyperglycemia reflects transient glycemic variability and disease severity, which can independently predicts Infarct volume, END risk, and 90‐day functional disability (Seners et al. 2015).

ROC analysis was employed to assess the diagnostic performance of the TyG index, GAR, and their combination in IAPI patients. The TyG index demonstrated an AUC of 0.769 (95% CI: 0.657‐0.841) for predicting END in BAD patients, with an optimal cutoff threshold of 8.52 (sensitivity 74.2%, specificity 80.3%). Similarly, GAR achieved an AUC of 0.752 (95% CI: 0.632‐0.826) at the cutoff of 18.72 (sensitivity 80.8%, specificity 78.6%). The combined model exhibited superior discriminative capacity (AUC = 0.839, 95% CI: 0.728‐0.882), outperforming individual biomarkers (DeLong's test: *p* < 0.05 vs. TyG; *p* < 0.01 vs. GAR). Both biomarkers are readily available through standard fasting blood tests, requiring only triglyceride, glucose, and HbA1c measurements. Clinically elevated TyG index (> 8.5) and GAR (> 18.7) should prompt intensified neurological monitoring (e.g., hourly NIHSS assessments) and consideration of early neuroprotective interventions.

In conclusion, the TyG index and GAR serve as independent predictors of END in IAPI (adjusted OR = 3.396 and 2.214, respectively, both *p* < 0.01), as validated by multivariable regression analysis. The biomarkers demonstrate moderate individual diagnostic accuracy, while their synergistic use significantly enhances predictive power. These cost‐effective biomarkers enable risk stratification that may guide (1) early rehabilitation protocols. (2) personalized secondary prevention strategies. (3) Prognostic communication with families.

Our study had several limitations. (1) Single‐center observational design introduces potential selection bias (e.g., referral bias). (2) Lack of external validation in multiethnic populations. (3) Absence of continuous glucose monitoring data.

## Author Contributions

**Jian Ge**: methodology, writing – original draft, and investigation. **Chunjie Song**: funding acquisition, writing – original draft, writing – review and editing. **Yuanyuan Han**: writing – review and editing, project administration, investigation, validation, supervision, and conceptualization.

## Conflicts of Interest

The authors declare no conflicts of interest.

## Peer Review

The peer review history for this article is available at https://publons.com/publon/10.1002/brb3.70746.

## Data Availability

The data that support the findings of this study are available from the corresponding author upon reasonable request.

## References

[brb370746-bib-0001] Batiha, G. E. , H. M. Al‐Kuraishy , and T. J. Al‐Maiahy , et al. 2022. “Plasminogen Activator Inhibitor 1 and Gestational Diabetes: The Causal Relationship.” Diabetology and Metabolic Syndrome 14, no. 1: 127. 10.1186/s13098-022-00900-2. 36076264 PMC9454110

[brb370746-bib-0002] Bi, X. , X. Liu , and J Cheng . 2021. “Monocyte to High‐Density Lipoprotein Ratio is Associated With Early Neurological Deterioration in Acute Isolated Pontine Infarction.” Front Neurol 12: 678884. 10.3389/fneur.2021.678884. 34262524 PMC8273253

[brb370746-bib-0003] Capes, S. E. , D. Hunt , K. Malmberg , and H. C Gerstein . 2000. “Stress Hyperglycaemia and Increased Risk of Death After Myocardial Infarction in Patients With and Without Diabetes: A Systematic Overview.” Lancet 355, no. 9206: 773–778. 10.1016/S0140-6736(99)08415-9. 10711923

[brb370746-bib-0004] Capes, S. E. , D. Hunt , K. Malmberg , P. Pathak , and H. C Gerstein . 2001. “Stress Hyperglycemia and Prognosis of Stroke in Nondiabetic and Diabetic Patients: A Systematic Overview.” Stroke; A Journal of Cerebral Circulation 32, no. 10: 2426–2432. 10.1161/hs1001.096194. 11588337

[brb370746-bib-0005] Caplan, L. R. 1989. “Intracranial Branch Atheromatous Disease: A Neglected, Understudied, and Underused Concept.” Neurology 39, no. 9: 1246–1250. 10.1212/wnl.39.9.1246. 2671793

[brb370746-bib-0006] Chen, X. , Z. Liu , J. Miao , et al. 2019. “High Stress Hyperglycemia Ratio Predicts Poor Outcome After Mechanical Thrombectomy for Ischemic Stroke.” Journal of Stroke and Cerebrovascular Diseases 28, no. 6: 1668–1673. 10.1016/j.jstrokecerebrovasdis.2015.10.019. 30890395

[brb370746-bib-0007] Çoban, G. , E. Çifçi , E. Yildirim , and A. M Ağıldere . 2015. “Predisposing Factors in Posterior Circulation Infarcts: A Vascular Morphological Assessment.” Neuroradiology 57, no. 5: 483–489. 10.1007/s00234-015-1490-z. 25666230

[brb370746-bib-0008] Dungan, K. M. , S. S. Braithwaite , and J. C Preiser . 2009. “Stress Hyperglycaemia.” Lancet 373, no. 9677: 1798–1807. 10.1016/S0140-6736(09)60553-5. 19465235 PMC3144755

[brb370746-bib-0009] Enriquez‐Marulanda, A. , P. Amaya‐Gonzalez , and J. L Orozco . 2016. “Pontine Warning Syndrome: A Chameleon of Ischemic Stroke.” The Neurologist 21, no. 6: 93–96. 10.1097/NRL.0000000000000092. 27801767

[brb370746-bib-0010] Feng, X. , Y. Yao , L. Wu , C. Cheng , Q. Tang , and S Xu . 2022. “Triglyceride‐Glucose Index and the Risk of Stroke: A Systematic Review and Dose‐Response Meta‐Analysis.” Hormone and Metabolic Research 54, no. 3: 175–186. 10.1055/a-1766-0202. 35276743

[brb370746-bib-0011] Gökçal, E. , E. Niftaliyev , G. Baran , Ç. Deniz , and T Asil . 2017. “Progressive Deficit in Isolated Pontine Infarction: The Association With Etiological Subtype, Lesion Topography and Outcome.” Acta Neurologica Belgica 117, no. 3: 649–654. 10.1007/s13760-017-0827-2. 28776182

[brb370746-bib-0012] Huang, R. , X. Zhang , W. Chen , J. Lin , Z. Chai , and X Yi . 2016. “Stroke Subtypes and Topographic Locations Associated With Neurological Deterioration in Acute Isolated Pontine Infarction.” Journal of Stroke and Cerebrovascular Diseases 25, no. 1: 206–213. 10.1016/j.jstrokecerebrovasdis.2015.10.019. 26508683

[brb370746-bib-0013] Kim, J. H. , and Y Do . 2023. “An Isolated Pontine Infarct Extending to the Basal Pontine Surface has a Higher Abnormal Ankle‐brachial Index.” Medicine (Baltimore) 102, no. 52: e36829. 10.1097/MD.0000000000036829. 38206713 PMC10754564

[brb370746-bib-0014] Kleindorfer, D. O. , A. Towfighi , S. Chaturvedi , et al. 2021. “2021 Guideline for the Prevention of Stroke in Patients With Stroke and Transient Ischemic Attack: A Guideline From the American Heart Association/American Stroke Association.” Stroke 52, no. 7: e364–e467. 10.1161/STR.0000000000000375. 34024117

[brb370746-bib-0015] Kosmas, C. E. , M. D. Bousvarou , C. E. Kostara , E. J. Papakonstantinou , E. Salamou , and E Guzman . 2023. “Insulin Resistance and Cardiovascular Disease.” Journal of International Medical Research 51, no. 3: 3000605231164548. 10.1177/03000605231164548. 36994866 PMC10069006

[brb370746-bib-0016] Lee, M. , C. H. Kim , Y. Kim , et al. 2021. “High Triglyceride Glucose Index is Associated With Poor Outcomes in Ischemic Stroke Patients After Reperfusion Therapy.” Cerebrovascular Diseases 50, no. 6: 691–699. 10.1159/000516950. 34229319

[brb370746-bib-0017] Lemkes, B. A. , J. Hermanides , J. H. Devries , F. Holleman , J. C. Meijers , and J. B Hoekstra . 2010. “Hyperglycemia: A Prothrombotic Factor?” Journal of Thrombosis and Haemostasis 8, no. 8: 1663–1669. 10.1111/j.1538-7836.2010.03910.x. 20492456

[brb370746-bib-0018] Liu, M. , X. Yang , Y. Jiang , et al. 2024. “The Role of Triglyceride‐glucose Index in the Differential Diagnosis of Atherosclerotic Stroke and Cardiogenic Stroke.” BMC Cardiovascular Disorders [Electronic Resource] 24, no. 1: 295. 10.1186/s12872-024-03857-4. 38851694 PMC11162012

[brb370746-bib-0019] Liu, Y. , H. Peng , J. Wang , et al. 2022. “Risk Factors for Early Neurological Deterioration in Acute Isolated Pontine Infarction Without any Causative Artery Stenosis.” BMC Neurology [Electronic Resource] 22, no. 1: 332. 10.1186/s12883-022-02861-5. 36057555 PMC9440546

[brb370746-bib-0020] Ma, X. , Y. Han , L. Jiang , and M Li . 2022. “Triglyceride‐Glucose Index and the Prognosis of Patients With Acute Ischemic Stroke: A Meta‐Analysis.” Hormone and Metabolic Research 54, no. 6: 361–370. 10.1055/a-1853-9889. 35697045

[brb370746-bib-0021] Maeshima, S. , A. Osawa , Y. Miyazaki , H. Takeda , and N Tanahashi . 2012. “Functional Outcome in Patients With Pontine Infarction After Acute Rehabilitation.” Neurological Sciences 33, no. 4: 759–764. 10.1007/s10072-011-0812-0. 21979558

[brb370746-bib-0022] Merlino, G. , S. Pez , R. Sartor , et al. 2023. “Stress Hyperglycemia as a Modifiable Predictor of Futile Recanalization in Patients Undergoing Mechanical Thrombectomy for Acute Ischemic Stroke.” Frontiers in Neurology 14: 1170215. 10.3389/fneur.2023.1170215. 37273693 PMC10235599

[brb370746-bib-0023] Merlino, G. , S. Pez , Y. Tereshko , et al. 2022. “Stress Hyperglycemia Does not Affect Clinical Outcome of Diabetic Patients Receiving Intravenous Thrombolysis for Acute Ischemic Stroke.” Frontiers in Neurology 13: 903987. 10.3389/fneur.2022.903987. 35769366 PMC9234697

[brb370746-bib-0024] Merlino, G. , M. Romoli , R. Ornello , et al. 2024. “Stress Hyperglycemia Is Associated With Futile Recanalization in Patients With Anterior Large Vessel Occlusion Undergoing Mechanical Thrombectomy.” European Stroke Journal 9, no. 3: 613–622. 10.1177/23969873241247400. 38624043 PMC11418448

[brb370746-bib-0025] Mi, D. , Y. Wang , Y. Wang , and L Liu . 2020. “Insulin Resistance is an Independent Risk Factor for Early Neurological Deterioration in Non‐diabetic Patients With Acute Ischemic Stroke.” Neurological Sciences 41, no. 6: 1467–1473. 10.1007/s10072-019-04221-7. 31938983

[brb370746-bib-0026] Oh, S. , O. Y. Bang , C. S. Chung , K. H. Lee , W. H. Chang , and G. M Kim . 2012. “Topographic Location of Acute Pontine Infarction is Associated With the Development of Progressive Motor Deficits.” Stroke; A Journal of Cerebral Circulation 43, no. 3: 708–713. 10.1161/STROKEAHA.111.632307. 22343639

[brb370746-bib-0027] Palacio, S. , L. A. McClure , O. R. Benavente , C. Bazan 3rd , P. Pergola , and R. G Hart . 2014. “Lacunar Strokes in Patients With Diabetes Mellitus: Risk Factors, Infarct Location, and Prognosis: The Secondary Prevention of Small Subcortical Strokes Study.” Stroke; A Journal of Cerebral Circulation 45, no. 9: 2689–2694. 10.1161/STROKEAHA.114.005018. PMC4146755 25034716

[brb370746-bib-0028] Pan, Y. , X. Cai , J. Jing , et al. 2017. “Stress Hyperglycemia and Prognosis of Minor Ischemic Stroke and Transient Ischemic Attack: The CHANCE Study (Clopidogrel in High‐Risk Patients With Acute Nondisabling Cerebrovascular Events).” Stroke 48, no. 11: 3006–3011. 10.1161/STROKEAHA.117.019081. 29051218

[brb370746-bib-0029] Qu, L. , S. Fang , Z. Lan , et al. 2024. “Association Between Atherogenic Index of Plasma and New‐onset Stroke in Individuals With Different Glucose Metabolism Status: Insights From CHARLS.” Cardiovascular Diabetology 23, no. 1: 215. 10.1186/s12933-024-02314-y. 38907337 PMC11193183

[brb370746-bib-0030] Roberts, G. W. , S. J. Quinn , and N. Valentine , et al. 2015. “Relative Hyperglycemia, a Marker of Critical Illness: Introducing the Stress Hyperglycemia Ratio.” Journal of Clinical Endocrinology and Metabolism 100, no. 12: 4490–4497. 10.1210/jc.2015-2660. 26485219

[brb370746-bib-0031] Scott, D. A. , C. Ponir , M. D. Shapiro , and P. A Chevli . 2024. “Associations Between Insulin Resistance Indices and Subclinical Atherosclerosis: A Contemporary Review.” American Journal of Preventive Cardiology 18: 100676. 10.1016/j.ajpc.2024.100676. 38828124 PMC11143894

[brb370746-bib-0032] Seners, P. , G. Turc , C. Oppenheim , and J. C Baron . 2015. “Incidence, Causes and Predictors of Neurological Deterioration Occurring Within 24 h Following Acute Ischaemic Stroke: A Systematic Review With Pathophysiological Implications.” Journal of Neurology, Neurosurgery, and Psychiatry 86, no. 1: 87–94. 10.1136/jnnp-2014-308327. 24970907

[brb370746-bib-0033] Snarska, K. K. , H. Bachórzewska‐Gajewska , K. Kapica‐Topczewska , et al. 2017. “Hyperglycemia and Diabetes Have Different Impacts on Outcome of Ischemic and Hemorrhagic Stroke.” Archives of Medical Science 13, no. 1: 100–108. 10.5114/aoms.2016.61009. 28144261 PMC5206364

[brb370746-bib-0034] Takahashi, Y. , T. Yamashita , R. Morihara , et al. 2017. “Different Characteristics of Anterior and Posterior Branch Atheromatous Diseases With or Without Early Neurologic Deterioration.” Journal of Stroke and Cerebrovascular Diseases 26, no. 6: 1314–1320. 10.1016/j.jstrokecerebrovasdis.2015.10.019. 28365073

[brb370746-bib-0035] Tremblay, A. J. , B. Lamarche , C. F. Deacon , S. J. Weisnagel , and P Couture . 2014. “Effects of Sitagliptin Therapy on Markers of Low‐grade Inflammation and Cell Adhesion Molecules in Patients With Type 2 Diabetes.” Metabolism 63, no. 9: 1141–1148. 10.1016/j.metabol.2014.06.004. 25034387

[brb370746-bib-0036] Virani, S. S. , A. Alonso , E. J. Benjamin , et al; 2020. “American Heart Association Council on Epidemiology and Prevention Statistics Committee and Stroke Statistics Subcommittee. Heart Disease and Stroke Statistics‐2020 Update: A Report From the American Heart Association.” Circulation 141, no. 9: e139–e596. 10.1161/CIR.0000000000000757. 31992061

[brb370746-bib-0037] Wang, L. , Q. Cheng , T. Hu , et al. 2022. “Impact of Stress Hyperglycemia on Early Neurological Deterioration in Acute Ischemic Stroke Patients Treated With Intravenous Thrombolysis.” Frontiers in Neurology 13: 870872. 10.3389/fneur.2022.870872. 35645975 PMC9136409

[brb370746-bib-0038] Wang, Y. , G. Jiang , J. Zhang , J. Wang , W. You , and J Zhu . 2023. “Blood Glucose Level Affects Prognosis of Patients Who Received Intravenous Thrombolysis After Acute Ischemic Stroke? A Meta‐Analysis.” Frontiers in Endocrinology (Lausanne) 14: 1120779. 10.3389/fendo.2023.1120779. PMC10130663 37124754

[brb370746-bib-0039] Yamamoto, Y. , T. Ohara , M. Hamanaka , et al. 2010. “Predictive Factors for Progressive Motor Deficits in Penetrating Artery Infarctions in Two Different Arterial territories.” Journal of the Neurological Sciences 288, no. 1‐2: 170–174. 10.1016/j.jns.2009.08.065. 19836756

[brb370746-bib-0040] Yao, M. , Y. Hao , T. Wang , et al. 2023. “A Review of Stress‐induced Hyperglycaemia in the Context of Acute Ischaemic Stroke: Definition, Underlying Mechanisms, and the Status of Insulin Therapy.” Frontiers in Neurology 14: 1149671. 10.3389/fneur.2023.1149671. 37025208 PMC10070880

[brb370746-bib-0041] Yuan, C. , S. Chen , Y. Ruan , et al. 2021. “The Stress Hyperglycemia Ratio Is Associated With Hemorrhagic Transformation in Patients With Acute Ischemic Stroke.” CIA 16: 431–442. 10.2147/CIA.S280808. PMC7955757 33727806

[brb370746-bib-0042] Zhou, X. , C. Kang , Y. Hu , and X Wang . 2023. “Study on Insulin Resistance and Ischemic Cerebrovascular Disease: A Bibliometric Analysis via CiteSpace.” Frontiers in Public Health 11: 1021378. 10.3389/fpubh.2023.1021378. 36950100 PMC10025569

[brb370746-bib-0043] Zhou, Y. , Y. Pan , H. Yan , et al. 2020. “Triglyceride Glucose Index and Prognosis of Patients With Ischemic Stroke.” Frontiers in Neurology 11: 456. 10.3389/fneur.2020.00456. 32587566 PMC7297915

[brb370746-bib-0044] Zhu, J. , Y. Li , Y. Wang , S. Zhu , and Y Jiang . 2022. “Higher Prevalence of Diabetes in Pontine Infarction Than in Other Posterior Circulation Strokes.” Journal of Diabetes Research 2022: 4819412. 10.1155/2022/4819412. 35127950 PMC8813299

